# Predictors of UK postgraduate researcher attendance behaviours and mental health-related attrition intention

**DOI:** 10.1007/s12144-022-04055-1

**Published:** 2022-12-08

**Authors:** Clio Berry, Jeremy E. Niven, Cassie M. Hazell

**Affiliations:** 1 Primary Care and Public Health, Brighton and Sussex Medical School, Watson Building, Falmer, BN1 9PH UK; 2 School of Life Sciences, University of Sussex, Falmer, UK; 3 School of Psychology, University of Westminster, London, UK

**Keywords:** Doctoral student, Mental health, Perfectionism, Supervision, Attrition

## Abstract

High rates of postgraduate researchers (PGRs) terminate their studies early. This attrition can have detrimental personal consequences, and results in a loss of productivity, and research and innovation for the higher education sector and society as a whole. PGRs are vulnerable to the experience of mental health problems; a factor that appears to be increasing attrition amongst students in the UK. However, investigation of the determinants of problems with PGRs’ attendance and influencing intention to discontinue their studies is rare. Here, we consider the relative predictive validity of a set of putative predictors (mental health symptoms, demographic, occupational, psychological, social, and relational) of attendance behaviours (absenteeism, presenteeism, mental health-related intermission) and early attrition intention amongst UK PGRs. Depression, anxiety, and suicidality predicted attendance behaviours and greater attrition intention. Individual demographic and occupational factors predicted all outcomes. Psychological, social and relational factors had less predictive validity, although individual variables in these conceptual clusters did significantly predict some outcomes. Our results suggest that interventions to reduce high rates of mental health problems are likely to improve attendance behaviours, and reduce the extent to which PGRs intermit or consider ending their PhD studies for mental health-related reasons. Initiatives designed to improve supervisory relationships and reduce loneliness may also reduce absenteeism, intermission and attrition intention.

## Introduction

Doctoral attrition is high in many countries, with reported rates of up to 40 to 50% of postgraduate researchers (PGRs) terminating their PhD studies before completion (Geven et al., 2017; Litalien & Guay, 2015). Attrition can be considered a process, in which PGRs weigh the costs and benefits of persisting or discontinuing, and then do or do not actually end their studies accordingly (Jaksztat et al., 2021). Attrition may be provoked by reasons outside of the PhD, such as other opportunities, changing goals, or family obligations (Maher et al., 2017), and is not in itself ‘bad’. However, for many PGRs, attrition results in adverse psychological, financial and employment outcomes, as well as there being problematic consequences of non-completed PhD research for supervisors, institutions, and society (Litalien & Guay, 2015).

There has been limited research attention to PGR attrition (Jaksztat et al., 2021; Litalien & Guay, 2015), especially as it relates to mental health problems and other psychological factors (Jaksztat et al., 2021). The few known studies that have been conducted in this area originate from the US, where the PhD experience is unique; for example, typically being more structured and longer in duration (Jaksztat et al., 2021). This lack of research is especially concerning as increasing numbers of university students generally in the UK appear to be discontinuing their studies due to poor mental health (The Guardian, 2017).

Much evidence has emerged in the past few years in particular to suggest that PGRs experience high rates of stress (Hazell et al., 2020), depression, anxiety and suicidality; seemingly at rates that exceed those seen in other student and working populations (Hazell et al., 2021; Levecque et al., 2017). The PhD itself and its surrounding environment have been implicated in the onset and exacerbation of PGR mental health problems (Berry et al., 2020; Levecque et al., 2017), with the recent COVID-19 pandemic seemingly exacerbating the poor mental health and wellbeing of the adult population generally (Amerio et al., 2021; Odone et al., 2020), and PGRs specifically (Byrom, 2020) even further.

Poor mental health and suicidality are inherently distressing and associated with negative health and functional outcomes (DeRoma et al., 2009; Okajima et al., 2015), including death by suicide; a frequently under-reported phenomenon (Visentin et al., 2019). A functional outcome for PGRs that is important, yet under-investigated, is mental health-related attrition. However, attrition is not the only index of disengagement from academic study. Disengagement can be conceptualised multi-dimensionally, with a focus on problematic attendance behaviours as well as attrition-related cognitions. Attendance behaviours themselves can be conceptualised as using multiple indices: absenteeism (non-planned/non-holiday absences); presenteeism (working or studying when unwell enough to take absence); and intermission (interruption or prolonged break from doctoral studies). Evidence suggests that PGRs with experience of mental health problems report greater absenteeism and presenteeism (Berry et al., 2021a), intermission, and intention to discontinue doctoral study (Castelló et al., 2017; González-Betancor & Dorta-González, 2020; Hunter & Devine, 2016).

The present study tests a comprehensive range of potential determinants of attendance behaviours and attrition intention amongst a large sample of UK PGRs. We were informed by our previous work which identified putative determinants of mental health symptoms as demographic, occupational, psychological, social and relational in nature (Berry et al., 2021b). Taking this same approach here aligns with studies that suggest influences on doctoral attrition are complex and multifactorial (Castelló et al., 2017; Gardner, 2010), and with theoretical models of student retention as being a product of both academic and social integration (Tinto, 2016). We first predicted that mental health symptoms would predict poorer attendance and attrition intention. 

We next predicted that demographic and occupational factors would predict attendance and attrition intention. White PGRs report fewer days absent but more severe presenteeism (Berry et al., 2021a). Female PGRs appear to spend more time in presenteeism and absenteeism (Berry et al., 2021a), report greater number of intermissions (Moore & Keith, 1992), greater attrition intention (Castelló et al., 2017), and are more likely to actually discontinue their PhD studies (Jaksztat et al., 2021). A lack of funding is associated with greater attrition intention (Castelló et al., 2017) and attrition (Litalien & Guay, 2015). PGRs who spend less time per week in PhD study report greater attrition intention (Castelló et al., 2017). However, it is unclear to what extent demographic and PhD-study related characteristics independently and uniquely influence attendance behaviours and attrition intention, i.e. when modelled simultaneously and when considering psychological and social factors.

Finally, we predicted that psychological, social and relationship factors would predict PGR attendance behaviours and attrition intention. With respect to psychological factors, PGRs who perceive themselves to lack competence report greater attrition intention (Castelló et al., 2017) and attrition (Litalien & Guay, 2015). Moreover, lower academic aspirations are associated with a greater number of intermissions (Moore & Keith, 1992). This suggests a potential role in doctoral attrition for the psychological traits of perfectionism (*i.e.* having high standards and/or believing one is not meeting their standards) and impostor thoughts (*i.e.* believing that one is not as competent as others perceive one to be). Moreover, the nature of interpersonal relationships in general, and specifically with the supervisor, are likely important. Social disconnectedness is associated with PGR attrition intention (Castelló et al., 2017; Volkert et al., 2018). Supervisory relationship stressors and lack of psychological support are associated with attrition intention (Litalien & Guay, 2015; Volkert et al., 2018), and clear authoritative direction, *i.e*. supervisor agency, seems important in doctoral completion (McCray & Joseph-Richard, 2020). Mental health problems may be a confounding factor here, however, as previous research has found supervisory relationship qualities to predict mental health symptoms (Berry et al., 2021b) and thus, this should be accounted for in modelling associations with attendance and attrition-related outcomes.

Research considering the relative contribution of influences across multiple domains on PGR disengagement is rare, especially considering indices spanning multiple proxies of attendance and attrition intention. Furthermore, although research has considered whether dissatisfaction is a precursor to attrition (González-Betancor & Dorta-González, 2020), no known study has tested whether problematic attendance behaviours themselves may function as precursors to attrition intention. We predicted that this would be the case because academic disengagement leads to intention to leave academia (Lesko & Corpus, 2006). Furthermore, absence and presenteeism reduce productivity (Johns, 2010), organisational commitment and embeddedness (Boswell et al., 2008), and additionally likely increase time-to-completion, which in turn predicts doctoral attrition (de Valero, 2001).

Based upon our predictions, we tested the specific hypotheses that attendance behaviours and attrition intention would be predicted by mental health symptoms (depression, anxiety, suicidality), and then by the following factors:

- demographic; age, gender, ethnicity, UK residency, disability and lifetime mental health problem prevalence,
- occupational; fulltime status, funding, year of study, fieldwork, time spent in occupational activity,
- psychological; impostor thoughts, perfectionistic standards and discrepancy,
- social; loneliness and multiple group memberships, and
- relational; supervisory relationship communion and agency.

## Materials and methods

### Participants and procedure

Data were obtained from a national online self-report survey (U-DOC) conducted in the UK between 2018 and 2019. The survey was designed to contain a battery of self-report survey assessments and qualitative data pertaining to PGR mental health symptoms, and multiple factors considered to be potential correlates of these symptoms and associated behaviours. Participants were a convenience sample of 3352 current PGRs who provided informed consent and then completed questionnaire measures and qualitative free-text questions. The participant inclusion criteria were that participants were aged 18 years or over and were currently studying for their PhD at a UK University. Participants were recruited by contacting all UK doctoral schools (*N* = 162) and asking for them to promote the study, via email, institutional communications and through social media advertising (e.g. Twitter, Facebook). The research team additionally promoted the study via social media platforms. The study received research ethics approval from the University of Sussex Sciences and Technology Cross-Schools Research Ethics Committee (Reference: ER/CH283/9). Additional methodological and sample details are reported elsewhere (Authors, 2021).

### Measures

#### Absenteeism and presenteeism

Absenteeism and presenteeism data were collected using items from the Institute for Medical Technology Assessment Productivity Cost Questionnaire (iMTA PCQ) – Presenteeism Scale (Bouwmans et al., 2015). For the present study, binary variables were used to indicate absenteeism (no absenteeism 0, absenteeism 1) or presenteeism (no presenteeism 0, presenteeism 1) specifically regarding PhD study in the past month, excluding planned annual leave or holidays. Absenteeism referred to days absent and presenteeism referred to “days in which you worked but during this time were bothered by physical or psychological problems”. Additional information about the measure of absenteeism and presenteeism has been published previously (Berry et al., 2021a).

#### Mental health-related intermission and attrition intention

Respondents indicated if they had had to take a break from their PhD studies for mental health-related reasons (mental health-related intermission), and if they had considered terminating their PhD studies for mental health-related reasons (mental health-related attrition intention). In both cases, respondents indicated whether statements were ‘true’ or ‘false’; coded as ‘1’ or ‘0’, respectively. A third option ‘not sure’ was also coded as ‘0’.

#### Mental health symptoms

The 9-item Patient Health Questionnaire (PHQ-9 (Kroenke et al., 2001)) was used to capture depression symptoms, the 7-item (GAD-7 (Spitzer et al., 2006) to capture anxiety symptoms, and the 4-item Suicide Behaviors Questionnaire – Revised (SBQ-R (Linehan & Nielsen, 1981)) to capture suicidality.

#### Demographic characteristics

Participants self-reported age in years, gender (coded for this study as female *versus* male/another identity), ethnicity (coded as White *versus* non-White), UK citizenship (*versus* non-UK citizenship), disability status (not including mental health problems), and lifetime prevalence of mental health problems (coded as pre-existing mental health problems up to and including during undergraduate studies *versus* onset during postgraduate study).

#### Occupational characteristics

Participants were asked to self-report their PhD study mode (fulltime *versus* part-time), funding (full, partial or self-funded), year of study, and past or planned fieldwork (*versus* none). Participants estimated how many hours per week on average they spent engaged in PhD study, teaching activities, and any other employment. These were summed to create weekly average of hours spent in occupational activity.

#### Psychological factors

Impostor thoughts were measured using the 20-item Clance Impostor Phenomenon Scale (CIPS (Clance, 1985)), and perfectionistic standards (*i.e.* high expectations for oneself) and discrepancy (*i.e.* the degree to which one thinks they fail to meet these expectations) using the 8-item Short Almost Perfect Scale (SAPS (Rice et al., 2014)).

#### Social factors

The social variables captured were loneliness (*i.e.* the subjective sense of deficiency in one’s social relationships), and multiple group memberships (*i.e.* the degree to which one perceives they have ties and relationships with multiple social groups). Loneliness was measured using the 20-item UCLA Loneliness Scale (Russell et al., 1978). Multiple group memberships was captured using a 4-item self-report scale derived from the Exeter Identity Transition Scale (Haslam et al., 2008).

#### Relational factors

The relational qualities of the supervisory relationship were measured using the 41-item Questionnaire on Supervisor–Doctoral student Interaction (QSDI (Mainhard et al., 2009)). Two dimensional scores were used; agency (influence and leadership) and communion (proximity and cooperativeness).

### Analysis

All analysis was conducted in SPSS (version 26.0). Bivariate associations between putative predictor variables, attendance behaviours and attrition intention were examined using t-test and chi-square models. Hierarchical logistic regression was used to test predictors of attendance behaviours and attrition intention in four separate models. Attendance behaviours and attrition intention were specified as binary categorical dependent variables in these models. Mental health symptom scores were added as predictors first, before then entering the demographic, occupational, psychological, social and relational factors in turn as separate blocks. Variables not showing bivariate associations with attendance behaviours and attrition intention were not entered. Hochberg’s correction was applied for multiple testing in the final models (Menyhart et al., 2021). These models met the requisite assumptions of the independence of errors and absence of significant multicollinearity. Moreover, with the exception of two standardised residuals in the presenteeism model and one in the attrition intention model, all standardised residuals were under 2.5. Cook’s distances and DFbetas all under 1, suggesting, showed no significant impact of unusual cases on any model. The Box-Tidwell test was used to confirm that the relationship between the logit (log-odds) of the outcome and each continuous predictor was linear. All interactions between the predictors and their logits were non-significant, with the exception of depression (PHQ-9), anxiety (GAD-7), and supervisory agency and communion (QSDI) in one model respectively. However, these interactions were not highly significant (p ≥ 0.03) and the sample size is large, therefore, we considered the assumption of non-linearity to be satisfied (Wuensch, 2021).

## Results

### Sample characteristics

Respondents were aged on average 30.74 (SD 8.82) years and 2205 (65.8%) were female. Overall, 1749 (52.2%) respondents identified as White British and 2114 (63.2%) as UK residents. In total, 1059 (31.8%) respondents reported having been given a diagnosis of a mental health disorder during their lifetime, and another 919 (27.6%) reported experiencing mental health problems with no formal diagnosis. The majority of students were fulltime (*n* = 2536, 81.4%) and had full (*n* = 2036, 65.4%) or partial funding (*n* = 413, 13.3%). Eight-hundred and thirty-four PGRs were in their first year (26.9%), 846 (27.3%) in their second, 756 (24.4%) in their third, 422 (13.6%) fourth, and 144 (4.6%) in the fifth year of PhD studies. A significant minority of students reported past (*n* = 767, 24.8%) or planned (*n* = 303, 9.8%) fieldwork. In total, 1069 (31.9%) PGRs reported taking non-planned/holiday absence in the past month and 1697 (50.6%) taking no absences. Overall, 1694 (50.5%) PGRs reported presenteeism in the past month, and 1201 (35.8%) reported none. In addition, 455 (13.6%) of PGRs had taken mental health-related intermission and 2604 (77.7%) had not (*i.e*. had rated this statement as false or not sure). Finally, 1097 (32.7%) of PGRs had considered ending their PhD studies for mental health-related reasons and 1963 (58.5%) had responded false or not sure. Ninety-seven PGRs (3.1%) in current continuation were removed from the dataset before analysis, for attendance behaviours and attrition intention were thought not to be equivalent in this context.

### Bivariate associations

Bivariate associations (Tables 1 and 2) demonstrated that past month absenteeism and presenteeism, having taken mental health-related intermission, and reporting mental health-related attrition intention, were all significantly associated with having a disability (Table 1), and with significantly greater depression, anxiety, suicidality, impostor thoughts, perfectionistic discrepancy, loneliness, and reduced supervisory communion (Table 2). Past month absence and intermission were associated with significantly reduced weekly occupational activity hours, whereas past month presenteeism and attrition intention were associated with more hours (Table 2). Absenteeism and presenteeism, and attrition intention, but not intermission, were significantly associated with younger age (Table 2) and being female (Table 1). Only absenteeism was associated with reduced perfectionistic standards, and only presenteeism and attrition intention were associated with higher standards (Table 2). Presenteeism, intermission and attrition intention were associated with being White (Table 1) and reduced perception of multiple group memberships (Table 2). Absenteeism was more likely for non-White and non-UK citizens (Table 1). Taking intermission and reporting attrition intention were more likely for UK citizens and people with more recent-onset mental health problems (Table 1). Presenteeism did not differ according to UK citizenship or pre-existing mental health problems (Table 1). Absenteeism, intermission and attrition intention, but not presenteeism, were associated with past or planned fieldwork (Table 1), and with reduced supervisory agency (Table 2). Full-time PGRs were more likely to report absenteeism and presenteeism, whereas a part-time mode was associated with taking intermission, with no association between study mode and attrition intention (Table 1). Absenteeism and presenteeism, and attrition intention, were more likely for fully funded PGRs and less likely for self-funded students (Table 1). Taking intermission was less likely for fully funded PGRs, and more likely in partial or self-funded modes (Table 1).

**Table 1:** Chi-square tests of associations between categorical study variables and attendance behaviours and attrition intention

Categorical variable		Any absence			Any presenteeism		Mental health–related intermission		Mental health–related attrition intention	
		Absence	No absence		Presenteeism	No presenteeism	Taken intermission	Not taken intermission	Considered ending	Not considered ending
Demographic predictors										
Gender	Female N(%)	**1090(59.9)**	**731(40.1)**		**1194(62.7)**	**709(37.3)**	300(14.9)	1710(85.1)	**759(37.7)**	**1252(62.3)**
	Non–female N(%)	**333(35.7)**	**599(64.3)**		**489(50.0)**	**489(50.0)**	150(14.5)	883(85.5)	**331(32.0)**	**702(68.0)**
	χ(df)	5.06(1)*			43.18(1)***		0.09(1)		9.65(1)**	
Ethnicity	White N(%)	**833(37.1)**	**1411(62.9)**		**1399(60.0)**	**932(40.0)**	**388(15.8)**	**2062(84.2)**	**937(38.2)**	**1514(61.8)**
	Non–White N(%)	**225(45.5)**	**270(54.5)**		**280(52.6)**	**252(47.4)**	**61(10.7)**	**509(89.3)**	**148(26.0)**	**422(74.0)**
	χ(df)	11.88(1)**			9.74(1)**		9.63(1)**		30.22(1)***	
UK citizenship	UK citizen N(%)	**627(35.6)**	**1136(64.4)**		1096(59.8)	737(40.2)	**316(16.5)**	**1603(83.5)**	**768(40.0)**	**1151(60.0)**
	Non–UK citizen N(%)	**442(44.1)**	**560(55.9)**		598(56.4)	463(43.6)	**139(12.2)**	**1000(87.8)**	**329(28.9)**	**811(71.1)**
	χ(df)	19.68(1)***			3.26(1)		10.23(1)**		38.73(1)***	
Disability^a^	Disability N(%)	**149(47.9)**	**162(52.1)**		**242(75.6)**	**78(24.4)**	**89(25.9)**	**254(74.1)**	**160(46.6)**	**183(53.4)**
	No disability N(%)	**878(37.2)**	**1482(62.8)**		**1398(56.5)**	**1077(43.5)**	**350(13.4)**	**2264(86.6)**	**900(34.4)**	**1715(65.6)**
	χ(df)	13.31(1)***			42.81(1)***		37.83(1)***		19.73(1)***	
Pre–existing mental health problems	Pre–existing M(SD)	528(42.1)	727(57.9)		940(71.8)	369(28.2)	**258(18.9)**	**1107(81.1)**	**648(47.5)**	**717(52.5)**
	Recent onset M(SD)	109(44.0)	139(56.0)		202(75.7)	65(24.3)	**99(34.7)**	**186(65.3)**	**189(66.3)**	**96(33.7)**
	χ(df)	0.30(1)			1.64(1)		34.87(1)***		33.49(1)***	
Occupational predictors										
PhD mode	Part–time M(SD)	**159(30.8)**	**358(69.2)**		**291(54.0)**	**248(46.0)**	**119(21.1)**	**445(78.9)**	197(34.9)	368(65.1)
	Full–time M(SD)	**910(40.5)**	**1336(59.5)**		**1402(59.6)**	**951(40.4)**	**336(13.5)**	**2157(86.5)**	900(36.1)	1593(63.9)
	χ(df)	16.88(1)***			5.66(1)*		21.09(1)***		0.31(1)	
Funding	Full funding M(SD)	**742(40.7)**	**1081(59.3)**		**1165(61.4)**	**732(38.6)**	**254(12.7)**	**1750(87.3)**	**753(37.6)**	**1251(62.4)**
	Partial funding M(SD)	**156(42.7)**	**209(57.3)**		**215(56.4)**	**166(43.6)**	**73(18.1)**	**330(81.9)**	**143(35.4)**	**261(64.6)**
	Self–funded M(SD)	**171(29.6)**	**406(70.4)**		**314(51.0)**	**302(49.0)**	**128(19.6)**	**524(80.4)**	**201(30.8)**	**451(69.2)**
	t(df)	25.581(2)***			21.68(2)***		22.65(2)***		9.78(2)**	
Fieldwork	None M(SD)	**660(36.2)**	**1164(63.8)**		1102(58.0)	797(42.0)	**247(12.4)**	**1753(87.6)**	**687(34.3)**	**1314(65.7)**
	Past/planned M(SD)	**409(43.5)**	**531(56.5)**		592(59.6)	402(40.4)	**208(19.7)**	**850(80.3)**	**409(38.7)**	**649(61.3)**
	χ(df)	14.04(1)***			0.63(1)		29.19(1)***		5.63(1)*	
Past month absenteeism	Absence	–		–	–	–	–	–	**455(42.6)**	**614(57.4)**
	No absence	–		–	–	–	–	–	**518(30.5)**	**1178(69.5)**
	χ(df)	–			–		–		**–**	
Past month presenteeism	Presenteeism	–		–	–	–	–		**788(46.5)**	**906(53.5)**
	No presenteeism	–		–	–	–	–		**241(20.1)**	**959(79.9)**
	χ(df)	–			–		–		214.20(1)***	
Mental health–related intermission	Intermission	–		–	–	–	–		**330(30.1)**	**125(6.4)**
	No intermission	–		–	–	–	–		**767(69.9)**	**1837(93.6)**
	χ(df)	–			–		–		312.42(1)***	

Significant associations are identified in bold

*** *p* < 0.001, ** *p* < 0.01, * *p* < 0.05. ^a^Excluding mental health problems

**Table 2:** T-tests and ANOVAs of associations between continuous study variables and attendance behaviours and attrition intention

Continuous variable		Any absence		Any presenteeism		Mental health–related intermission		Mental health–related attrition intention	
		Absence	No absence	Presenteeism	No presenteeism	Taken intermission	Not taken intermission	Considered ending	Not considered ending
Mental health symptoms									
Depression (PHQ–9)	M(SD)	**10.56(6.45)**	**7.80(6.20)**	**10.97(6.33)**	**6.28(5.43)**	**12.09(6.63)**	**8.58(6.24)**	**12.70(6.38)**	**7.10(5.50)**
	t(df)	–10.34(2201.29)***		–21.36(2785.73)***		–10.43(591.85)***		–24.21(1960.46)***	
Anxiety (GAD–7)	M(SD)	**9.70(5.41)**	**8.00(5.54)**	**10.37(5.36)**	**6.29(4.87)**	**11.03(5.52)**	**8.34(5.47)**	**11.39(5.38)**	**7.26(5.09)**
	t(df)	–7.90(2761.00)***		–21.25(2717.88)***		–9.61(3030.00)***		–20.65(2137.44)***	
Suicidality (SBQ–R)	M(SD)	**6.50(3.61)**	**5.68(3.23)**	**6.74(3.64)**	**4.96(2.72)**	**7.49(4.04)**	**5.74(3.22)**	**7.55(3.86)**	**5.17(2.80)**
	t(df)	–6.08(2024.96)***		–14.71(2763.45)***		–8.26(493.80)***		–16.92(1521.82)***	
Demographic predictors									
Age	M(SD)	**29.18(7.24)**	**31.55(9.60)**	**30.18(8.02)**	**31.34(9.87)**	31.13(8.46)	30.49(8.78)	**30.06(7.83)**	**30.88(9.18)**
	t(df)	7.37(2676.41)***		3.37(2239.52)*		–1.43(3057.00)		2.60(2577.17)*	
Occupational predictors									
Year of PhD study	M(SD)	2.17(1.27)	2.18(1.28)	**2.24(1.29)**	**2.11(1.26)**	**2.79(1.37)**	**2.10(1.25)**	**2.52(1.38)**	**2.03(1.20)**
	t(df)	0.11(2008.00)		–2.27(1950.04)*		–8.23(386.15)***		–8.35(1353.72)***	
Occupational activity hours	M(SD)	**39.41(15.23)**	**42.55(15.05)**	**42.79(14.76)**	**38.67(15.65)**	**39.29(16.74)**	**41.52(15.15)**	**42.67(15.67)**	**40.34(15.22)**
	t(df)	5.30(2732.00)***		–7.18(2860.00)***		2.64(582.92)**		–4.01(3024.00))***	
Psychological predictors									
Impostor thoughts (CIPS)	M(SD)	**71.62(15.630**	**67.54(16.50)**	**72.78(15.67)**	**63.93(15.74)**	**72.80(15.31)**	**68.46(16.34)**	**75.65(14.92)**	**65.59(15.90)**
	t(df)	–6.36(2674.00)***		**–14.65(2769.00)**		–5.20(569.92)***		–16.52(2080.10)***	
Perfectionism standards (SAPS–S)	M(SD)	**23.62(4.37)**	**23.97(4.09)**	**24.30(3.99)**	**23.09(4.45)**	23.92(4.35)	23.78(4.20)	**24.14(4.17)**	**23.61(4.24)**
	t(df)	2.06(2096.60)*		–7.37(2318.24)***		–0.64(2768.00)		–3.16(2768.00)**	
Perfectionism discrepancy (SAPS–D)	M(SD)	**20.63(5.44)**	**19.33(5.81)**	**20.95(5.37)**	**18.22(5.71)**	**21.44(5.20)**	**19.53(5.71)**	**21.94(4.86)**	**18.66(5.76)**
	t(df)	–5.85(2316.27)***		–12.74(2396.90)***		–6.73(581.71)***		–15.84(2280.38)***	
Social predictors									
Loneliness (UCLA)	M(SD)	**26.99(14.53)**	**23.01(14.63)**	**27.46(14.38)**	**20.64(14.27)**	**30.53(14.42)**	**23.66(14.54)**	**30.35(14.38)**	**21.55(13.96)**
	t(df)	–6.98(2759.00)***		–12.60(2888.00)***		–9.07(2905.00)***		–16.10(2905.00)***	
Multiple group memberships (MGM)	M(SD)	13.23(5.90)	13.26(6.11)	**12.93(6.06)**	**13.56(5.96)**	**12.51(6.02)**	**13.27(6.03)**	**12.22(6.04)**	**13.67(5.96)**
	t(df)	0.13(2763.00)		2.78(2892)**		2.41(2930.00)*		6.29(2930.00)***	
Relational predictors									
Supervisor relationship agency (QSDI–A)	M(SD)	**0.03(0.13)**	**0.06(0.13)**	0.04(0.13)	0.05(0.12)	**0.03(0.14)**	**0.05(0.12)**	**0.04(0.14)**	**0.05(0.12)**
	t(df)	4.43(2715.00)***		1.59(2689.90)		2.92(525.02)**		2.81(1775.09)**	
Supervisor relationship communion (QSDI–C)	M(SD)	**0.46(0.37)**	**0.52(0.34)**	**0.46(0.37)**	**0.54(0.32)**	**0.39(0.40)**	**0.51(0.34)**	**0.36(0.40)**	**0.57(0.30)**
	t(df)	4.68(2123.21)***		6.01(2733.52)***		6.16(518.87)***		13.99(1610.19)***	

Significant differences are identified in bold

*** *p* < 0.001, ** *p* < 0.01, * *p* < 0.05

### Multivariate predictors of attendance behaviours and attrition intention

The hierarchical logistic regression models (Table 3) showed that the predictor blocks explained significant variance in attendance behaviours and attrition intention, with some exceptions. First, social factors did not explain significant variance in any model, although this was marginal for the intermission model, in which loneliness was a significant predictor of intermission likelihood. Secondly, psychological and relational predictors did not explain significant variance in presenteeism or intermission. With respect to significant individual predictors when all blocks had been entered, past month absence (Table 3, model A) was significantly predicted by greater depression, younger age, non-female gender, White ethnicity, UK citizenship, not having a disability, not being self-funded, reduced weekly occupational hours, no fieldwork, and reduced supervisory agency. Past month presenteeism (Table 3, model B) was significantly predicted by greater depression and anxiety, being non-female and non-disabled, not being fully-funded, greater occupational weekly hours, and greater perfectionist standards. Having taken mental health-related intermission (Table 3, model C) was significantly predicted by greater anxiety, pre-existing mental health problems, more years of PhD study, reduced impostor thoughts, and greater perfectionistic discrepancy and loneliness. Mental health-related attrition intention (Table 3, model D) was predicted by greater depression and suicidality, being non-White, pre-existing mental health problems, more years of PhD study, reduced communion in the supervisory relationship, and not taking mental health related-intermission. All odds ratios reflected small size effects. The majority of individual predictors were significant at the respective Hochberg corrected alpha level (Table 3).

**Table 3:** Logistic regression models predicting absenteeism, presenteeism, mental health-related intermission and attrition intention

Model step	Parameter	Regression model A Past month absence		Regression model B Past month presenteeism		Regression model C Mental health–related intermission		Regression model D Mental health–related attrition intention	
		OR(B), [95% CI]	Wald	OR(B), [95% CI]	Wald	OR(B), [95% CI]	Wald	OR(B), [95% CI]	Wald
*Block 0 Symptoms*	Depression	1.05(0.05)***^a^**, [1.02, 1.07]**	13.30	1.06(0.06)***^b^**, [1.03, 1.10]**	**15.68**	0.98(–0.02), [0.94, 1.03]	0.55	1.08(0.07)***^c^**, [1.04, 1.12]**	**14.43**
	Anxiety	1.00(–0.01), [0.97, 1.02]	.174	1.07(0.06)***^b^**, [1.03, 1.10]**	**15.44**	1.01(0.06)*, [1.01, 1.11]	**4.80**	1.00(0.00), [0.96, 1.05]	0.01
	Suicidality	1.02(0.02), [0.99, 1.05]	1.83	1.09(0.08)***^b^**, [1.04, 1.13]**	**17.13**	1.04(0.04), [0.98, 1.09]	1.69	1.08(0.08)**^c^**, [1.03, 1.14]**	**10.85**
	*Chi–square (χ2(df))*	92.08(3)***		314.39(3)***		24.54(3)***		130.96(3)***	
	*Cox–Snell R*^*2*^	3.7%		15.4%		2.4%		12.7%	
Block 1 Demographic	Age	0.98(–0.02)**^a^**, [0.97, 0.99]**	**12.02**	1.00(0.00), [0.99, 1.01]	0.01	–	–	1.01(0.01), [0.99, 1.03]	1.73
	Female gender (0 non–female, 1 female)	0.74(–0.34)**^a^**, [0.61, 0.90]**	**9.56**	0.69(–0.37)**, [0.55, 0.87]	**10.20**	–	–	0.87(–0.14), [0.62, 1.23]	0.63
	White ethnicity (0 non–White, 1 White)	1.39(0.33)**, [1.10, 1.76]	**7.81**	0.81(–0.21), [0.62, 1.06]	2.37	0.90(–0.10), [0.55, 1.48]	0.17	0.58(–0.56)*, [0.37, 0.89]	**6.15**
	UK citizen (0 non–UK citizen, 1 UK citizen)	1.32(0.28)**^a^**, [1.09, 1.61]**	**8.14**	–	**–**	0.96(–0.04), [0.65, 1.42]	0.05	0.79 (–0.24), [0.56, 1.11]	1.86
	Disability (0 none, 1 disability)^a^	0.62(–0.471)**^a^**, [0.48, 0.82]**	**11.86**	0.53(–0.64)***^b^**, [0.37, 0.75]**	**13.08**	0.68(–0.39), [0.44, 1.06]	2.89	1.07(0.07), [0.71, 1.63]	0.11
	Pre–existing mental health problems (0 not pre–existing, 1 pre–existing)	–	–	–	–	1.73(0.55)*, [1.09, 2.74]	**5.39**	1.64(0.50)*, [1.05, 2.58]	**4.64**
	Chi–square (χ2(df))	65.26(5)***		38.62(4)***		22.50(4)***		30.98(6)***	
	Cox–Snell R^2^	6.3%		17.2%		4.6%		15.5%	
Block 2 Occupational	PhD mode (0 part–time, 1 fulltime)	0.90(–0.108), [0.68, 1.18]	0.60	1.12(0.11), [0.81, 1.54)	0.47	1.28(0.24), [0.79, 2.05]	1.01	–	–
	Year of PhD study	**–**	**–**	1.02(0.02), [0.94, 1.11]	0.24	1.46(0.38)***^b^**, [1.27, 1.69]**	**28.05**	1.23(0.21)**, [1.08, 1.41]	**9.37**
	Full funding (0 self/partial, 1 full)	1.03(0.03), [0.79, 1.35]	0.06	0.75(–0.29)*, [0.59, 0.95]	**5.65**	1.11(0.10), [0.66, 1.85]	0.15	0.72(–0.32), [0.51, 1.02]	3.35
	Self–funded (0 full/partial, 1 self)	0.58(–0.54)**^a^**, [0.42, 0.80]**	**11.12**	**–**	**–**	0.61(–0.49), [0.34, 1.10]	2.69	–	–
	Average weekly hours in occupation	0.98(–0.02)***^a^**, [0.98, 0.99]**	**31.93**	1.02(0.02)***^b^**, [1.01, 1.02]**	**15.41**	0.98(–0.20)**, [0.97, 0.99]	**11.05**	1.00(–0.00), [0.99, 1.01]	0.00
	Fieldwork (0 none, 1 past/planned)	0.69(–0.27)**^a^**, [0.64, 0.92]**	**8.09**	–	–	0.69(–0.37)*, [0.48, 0.97]	4.49	0.89(–0.11), [0.65, 1.23]	0.49
	Chi–square (χ2(df))	64.77(5)***		30.67(4)***		76.32(6)***		30.66(4)***	
	Cox–Snell R^2^	8.8%		18.5%		11.6%		18.1%	
Block 3 Psychological	Impostor thoughts (CIPS)	1.00(0.00), [0.99, 1.01]	0.25	1.01(0.01), [1.00, 1.02]	2.14	0.99(–0.01), [0.98, 1.01]	0.83	1.01(0.01), [1.00, 1.02]	2.04
	Perfectionism standards (SAPS–S)	0.98(–0.02), [0.95, 1.00]	3.75	1.04(0.03)*, [1.01, 1.06]	**5.58**	–	–	0.98(–0.02), [0.94, 1.02]	0.88
	Perfectionism discrepancy (SAPS–D)	1.02(0.02), [0.99, 1.05]	3.34	0.99(–0.01), [0.97, 1.02]	0.27	1.02(0.02), [0.97, 1.07]	0.65	1.03(0.03), [0.98, 1.07]	1.43
	Chi–square (χ2(df))	10.08(3)*		7.60(3)		0.70(2)		10.03(3)***	
	Cox–Snell R^2^	9.1%		18.8%		11.6%		19.0%	
Block 4 Social	Loneliness (UCLA)	1.00(0.00), [0.99, 1.01]	0.01	1.01(0.01), [0.99, 1.01]	1.03	1.02(0.02)*, [1.00, 1.03]	**4.00**	1.00(–0.00), [0.99, 1.01]	0.05
	Multiple group memberships (MGM)	–	–	1.01(0.01), [1.00, 1.03]	2.15	1.02(0.02), [0.99, 1.06]	2.35	1.01(0.01), [0.98, 1.03]	0.20
	Chi–square (χ2(df))	0.01(1)		2.75(2)		4.94(2)		0.37(2)	
	Cox–Snell R^2^	9.1%		19.0%		12.1%		19.0%	
Block 5 Relational	Supervisory relationship agency (QSDI–A)	0.42(–0.87)*, [0.21, 0.83]	**6.23**	–	–	0.34(–1.08), [0.09, 1.27]	2.59	0.80(–0.23), [0.24, 2.68]	0.14
	Supervisory relationship communion (QSDI–C)	0.86–0.16), [0.66, 1.11]	1.35	0.81(–0.21), [0.57, 1.15]	1.36	0.79(–0.23), [0.49, 1.27]	0.93	0.37(–0.99)***^c^**, [0.23, 0.59]**	**17.92**
	Chi–square (χ2(df))	7.51(2)*		1.37(1)		3.41(2)		19.77(2)***	
	Cox–Snell R^2^	9.4%		19.0%		12.4%		20.6%	
Block 6 Attendance behaviours	Past month absence	–	–	–	–	–	–	0.79(–0.24), [0.58, 1.07]	2.28
	Past month presenteeism	–	–	–	–	–	–	0.76(–0.27), [0.53, 1.09]	2.17
	Mental health–related intermission	–	–	–	–	–	–	0.30(–1.20)***^c^**, [0.20, 0.46]**	**32.42**
	Chi–square (χ2(df))	–		–		–		43.07(3)***	
	Cox–Snell R^2^	–		–		–		24.1%	

Significant associations are identified in bold

*** *p* < 0.001, ** *p* < 0.01, * *p* < 0.05. ^**a**^Significant according to Hochberg’s corrected level of *p* < 0.005 for absenteeism model, ^**b**^Significant according to Hochberg’s corrected level of *p* < 0.001 for presenteeism and intermission models, ^**c**^Significant according to Hochberg’s corrected level of *p* < 0.002 for attrition intention model. d– variable not entered as covariate, as not bivariately associated with dependent variables

## Discussion

This study aimed to test mental health symptoms, and demographic, occupational, psychological, social and relational factors as predictors of PGR attendance behaviours (absenteeism, presenteeism, mental health-related intermission) and attrition intention. Our study, using cross-sectional data, shows that demographic and occupational factors are significant predictors of PGR attendance behaviours (absenteeism, presenteeism, mental-heath-related intermission) and attrition intention. This was evident across all models (bivariate and multivariate), though specific demographic and occupational factors differed in their patterns of prediction. Psychological, social and relational factors had less predictive validity, but made significant contributions to some models. The largest effects according to individual odds ratios were for demographic and supervisory relationship characteristics, and additionally for mental health-related intermission as a predictor of attrition intention.

With respect to demographic and occupational characteristics, absenteeism was predicted in the multivariate model by being White and a UK citizen. One interpretation of this is that ethnically diverse and international students take fewer absences because they feel under greater pressure to be present and to succeed in their PhD research (Litalien & Guay, 2015); pressure that may be both socio-cultural and bureaucratic as related to visa status. Not taking absences may in turn contribute to distress, for non-White and non-UK citizens were found to be more likely to consider terminating their studies early for mental health reasons. More years of PhD study predicted greater likelihood of mental health-related intermission and attrition intention, even when controlling for current symptoms. Current findings suggest that demographic factors play a greater role in predicting attendance behaviours and attrition intention, compared to their seemingly smaller role in predicting PGR mental health symptoms *versus* psychological, social and relational factors (Berry et al., 2021b). It may be that demographic vulnerabilities have especially profound influence on behavioural outcomes. For example, socio-demographic characteristics influence the extent to which people experience stigma and discriminatory behaviours within academic institutions (Berry et al., 2021a);, factors that in turn influence academic disengagement (Casad et al., 2019). The associations with having a non-mental health disability were surprising. In the multivariate models, being disabled predicted reduced absenteeism and presenteeism, whereas in the bivariate associations, people with a disability reported greater presenteeism. This may reflect that the degree of flexibility provided by doctoral study, for example in working hours and locations, allows people with disabilities to work when best suits them, which reduces their absenteeism. With respect to presenteeism, mental health symptoms may be an additional explanatory factor. It might be that people with a disability report greater presenteeism mainly due to elevated mental health symptoms, and once these symptoms are covaried, this association reverses because this group can flexibly arrange their PhD study time around other health issues. This fits with the finding that PGRs feel less able to take absences for mental health compared to physical health reasons (Berry et al., 2021a).

Overall, mental health symptoms predicted greater absenteeism, presenteeism, intermission and attrition intention. Depression consistently predicted all outcomes except intermission, with anxiety predicting greater likelihood of presenteeism and intermission, and suicidality predicting greater likelihood of presenteeism and attrition intention. These findings support previous studies demonstrating that mental health problems result in greater absenteeism and presenteeism (Berry et al., 2021a), intermission (González-Betancor & Dorta-González, 2020), and intention to discontinue doctoral study (Castelló et al., 2017; Hunter & Devine, 2016). The predictive validity of depression is consistent with evidence that it predicts poor attendance and educational engagement, more so than anxiety and especially when persistent (Abu Ruz et al., 2018). That all symptoms predict presenteeism is intuitive, because presenteeism is defined as working when bothered by physical or psychological problems (Bouwmans et al., 2015). Regarding attrition intention, it seems likely that the co-influence of depression and suicidality here is related to hopelessness being implicated in both these problems (Beck et al., 2006; Labelle et al., 2013), and presumably in considering discontinuing PhD studies, especially in the absence of anticipated success. This aligns with qualitative data from the present sample that suggests suicidal ideation can occur in the context of PhD failure concerns, with suicide considered by some PGRs as potentially a more favourable hypothesised outcome than not completing their PhD (Authors, 2021). A previous study found that only the unique symptoms of anxiety, excluding those shared with depression, predicted educational attrition (Gorman et al., 2020). In the present study, this relationship was not observable for attrition intention, but anxiety alone uniquely predicted taking mental health-related intermission. It could be that anxious avoidance is the best predictor of taking intermission, with little independent role for symptoms of depression or suicidality. Alternatively, as current data are cross-sectional, the directionality of associations is not clear and it is possible that PGRs who have previously taken mental health-related intermission are then more anxious.

Whilst social factors were bivariately associated with attendance behaviours and attrition intention, loneliness and multiple group memberships contributed little to the prediction of attendance behaviours, with the exception of loneliness predicting mental health-related intermission. This is difficult to reconcile with prior research that suggests important roles for social and relational factors, albeit non-synonymous yet overlapping with those measured here. For example, it has been suggested that doctoral persistence is largely shaped by social interactions with peers and supervisors (Bean & Tinto, 1988; Litalien & Guay, 2015). Research evidence has additionally found that sense of belonging reduces attrition intention (van Rooij et al., 2019), and that the perceived institutional climate predicts time spent in absenteeism and presenteeism, and presenteeism severity (Berry et al., 2021a). It could be that social factors indirectly influence behavioural outcomes and attrition intention via symptomatology. If this is the case, social interventions should still reduce absenteeism, presenteeism, and mental health-related intermission and attrition intention, through improving symptoms. This is in keeping with the resilience protection model of doctoral completion (McCray & Joseph-Richard, 2020), which suggests that complex inter-relations between personal, environmental, professional and institutional factors influence successful completion.

Psychological factors showed little predictive validity for attendance behaviours and attrition intention, other than that PGRs with higher perfectionistic standards were more likely to engage in presenteeism. This contradicts a previous study which found perceived competence to be the central factor in explaining PhD attrition (Litalien & Guay, 2015). However, this previous study did not control for mental health symptoms. It could be that associations between psychological factors and attendance behaviours and attrition intention are again indirect via associations with mental health symptoms. Lower supervisory agency and communion respectively predicted greater absenteeism and mental health-related attrition intention, which is in keeping with evidence that supervision quality is associated with intent to discontinue PhD studies (van Rooij et al., 2019). Our findings therefore caution against the seemingly prevailing view that PGR wellbeing and success are determined by students’ individual competencies, with more limited roles for supervisory and institutional characteristics and actions (Sverdlik et al., 2018). Indeed, supervisors with PGRs who discontinue early due to mental health problems might benefit from specific attention as to the sense of communion characterising their supervisory relationships, and training and initiatives to help enhance this where necessary.

Finally, absenteeism and presenteeism were not predictive of attrition intention, yet mental health-related intermission did appear to be significantly protective. It could be that an intermission period enables PGRs to put into place supports to help scaffold their mental wellbeing, which helps them to feel able to continue their doctorate to completion. It may also be that PGRs who have not taken intermission reflect those who feel unable or unwilling to take a period of intermission, and are perhaps then more likely to consider discontinuing their studies completely. It is clear that in workplaces there are variable ‘absence cultures’, which encourage or discourage presenteeism (Ruhle & Süß, 2019). PGRs too endorse the existence of different absence cultures, enacted in individual supervisory relationships and at wider lab or department levels; influencing the extent to which PGRs feel able to take absences (Berry et al., 2021a). The current findings would suggest that absence cultures that create or reinforce reticence to take mental health-related intermission may actually increase attrition intention. It is important to consider, nonetheless, that intermission typically results in loss of income for fully-funded students, whereas absenteeism and presenteeism usually do not. Moreover, we acknowledge that current participants do not include PGRs who have discontinued their studies. Consequently, we do not know the nature of the association between having taken mental health-related intermission and later attrition. Nonetheless, intention to leave is considered one of the strongest predictors of attrition (Ertem & Gokalp, 2019), making it is plausible that mental health-related intermission protects against mental health-related attrition.

### Limitations and future directions

There are important limitations to note. The data used are cross-sectional. Therefore, regression analyses test whether associations modelled between variables are consistent with theorised directions of effects, and do not test the directionality or causality of these associations. Moreover, models tested include a large number of variables, which makes the unique contribution of individual covariates difficult to interpret (Kraha et al., 2012). In addition, the metric of the predictor variable influences the size of the odds ratios presented, for the odds ratio reflects the change in odds associated with a one-unit increase in the exposure (Szumilas, 2010). Therefore, odds ratios may be closer to one for symptoms, and psychological and social factors, because the unit of measurement is small compared to the size of any meaningful change. Whilst the Box-Tidwell test results for continuous predictor linearity were acceptable (Wuensch, 2021), it is possible that there was a slight degree of non-linearity that may have caused underestimation of effects of these predictors (Long, 2008). The risk of this with respect to depression and anxiety seems low, as these variables were significant in most models, yet it could be the case that the supervisory relationship is an even more powerful predictor of attendance behaviours than observed here.

The sample from which current data were derived is a self-selecting sample of UK PGRs and therefore, the generalisability of findings is constrained. This is a common challenge to research on PGRs, for their representation in epidemiological research is poor and they are often undifferentiated from other populations of students. We note that, overall, the sample is predominantly female, White, identified as UK citizens, and had full PhD funding in place. Efforts to engage male PhD students, those from minority ethnic backgrounds and those not of UK citizenship, without full funding, should be made to ensure greater representativeness of these groups. More specifically, the current sample reflects only current PGRs and not those who have discontinued their studies. Therefore, attrition intention and predictors thereof may actually correspond to PGRs who are less likely to actually terminate their PhD studies early. Future research should test longitudinal predictors of attendance, intermission, and attrition intention, and attrition itself. Finally, we have tested the specified predictors in these models independently, however, it seems likely that they interact. We would anticipate that psychological and social factors are mediated by their association with mental health symptoms, and that social and relational factors in addition are mediated by psychological factors, for example, supervisory relationships likely impact on PGRs’ perceived competence (Litalien & Guay, 2015).

There are several clear policy recommendations of the current study. Policy should mandate supervisor training regarding mental health disclosures and supporting students with the enablement of reasonable adjustments to mitigate the impact of mental health problems on their PhD engagement and attendance; supporting them with mental health-related intermission when necessary. Such training should additionally support supervisors to form effective relationships with PGRs, in which there is appropriate guidance, direction, proximity and support; whilst scaffolding the PGR to develop a sense of their own self-agency as an emerging researcher. Finally, institutions should be asked to ensure access to interventions for mental health symptoms, which are appropriate for and accessible to PGRs, and that help mitigate the impact of perfectionistic thinking. Moreover, institutions should be expected to examine their structures and processes and consider how these may promote connectedness, with the provision of social initiatives to increase social support and reduce loneliness.

### Conclusions

This study has identified a number of risk factors for absenteeism, presenteeism, and mental health-related intermission and attrition intention among UK PGRs. The most consistent predictive associations were that sociodemographic factors and mental health problems predicted attendance problems, intermission and attrition intention. Psychological and social factors made smaller and less robust contributions to the prediction of attendance and attrition intention, yet there appeared a role for perfectionism and loneliness in greater chance of presenteeism and taking intermission. Supervisory relationship quality appeared to reduce the likelihood of absenteeism and considering PhD attrition. Current findings emphasise the need to provide appropriate prevention and intervention initiatives for PGRs with mental health problems, including enhancement of social connectedness and supervisory relationship quality. Such initiatives should have dual benefits of reducing PGR mental health problems and scaffolding positive PhD attendance and completion intention.

## Data Availability

The dataset related to this study is available from the corresponding author upon reasonable request.
